# Supplementary material for: Amniotic Mesenchymal-Derived Extracellular Vesicles and Their Role in the Prevention of Persistent Post-Breeding Induced Endometritis
Source: Int J Mol Sci. 2023 Mar 8;24(6):5166. doi: 10.3390/ijms24065166 (PMC10049450; doi:10.3390/ijms24065166)
Supplement: Supplementary file 1 [file ijms-24-05166-s001.zip › ijms-2139497-supplementary.pdf]

Supplementary Files

Supplementary Table S1. Sperm motility parameters evaluated by CASA System of sperm treated at different time with different concentration of EVs, compared to CTR.

EV concentration ($x10^6$)/ml	VCL (μm/s)					VSL (μm/s)				
	0h	1h	2h	3h	4h	0h	1h	2h	3h	4h
**CTR**	76.34 ± 70.15	64.25 ± 3.19^{aA}	49.76 ± 4.21^{aB}	49.05 ± 4.77^{aB}	49.50 ± 5.80^{aB}	34.12 ± 39.47	29.31 ± 2.75^{aA}	20.05 ± 1.66^{aB}	20.57 ± 2.43^{aB}	19.46 ± 1.32^{aB}
**50**		64.89 ± 4.50^{aA}	52.88 ± 5.35^{aB}	49.74 ± 5.65^{aC}	47.02 ± 4.13^{aC}		10.23 ± 1.08^{bA}	25.44 ± 2.60^{bB}	24.95 ± 2.02^{bB}	19.02 ± 2.18^{aC}
**100**		60.66 ± 6.54^{aA}	52.15 ± 5.82^{aB}	74.89 ± 7.02^{bC}	36.64 ± 3.45^{bD}		26.14 ± 2.81^{cA}	22.49 ± 2.58^{aA}	35.18 ± 3.10^{bB}	16.10 ± 1.63^{bC}
**150**		56.36 ± 5.42^{aA}	83.22 ± 7.19^{bB}	101.95 ± 8.29^{cC}	65.70 ± 6.74^{bD}		18.74 ± 2.45^{dA}	37.96 ± 3.69^{cB}	36.28 ± 3.69^{dB}	23.12 ± 2.52^{cA}
**200**		66.01 ± 6.95^{aA}	69.11 ± 6.82^{cA}	47.54 ± 4.05^{aB}	42.64 ± 5.19^{bB}		19.55 ± 2.71^{dA}	25.18 ± 2.86^{bB}	21.27 ± 2.38^{aA}	17.51 ± 1.05^{aA}
**250**		52.55 ± 5.20^{aA}	71.14 ± 6.06^{cB}	50.96 ± 5.98^{aA}	42.26 ± 4.90^{bC}		19.21 ± 2.21^{dA}	30.82 ± 3.09^{dB}	27.14 ± 2.77^{bB}	14.65 ± 1.90^{bC}
**300**		75.94 ± 6.03^{bA}	40.55 ± 4.74^{dB}	56.05 ± 6.21^{aC}	69.83 ± 6.12^{cD}		31.61 ± 3.12^{aA}	14.21 ± 1.16^{eB}	27.05 ± 2.85^{bA}	24.53 ± 2.49^{cC}
**350**		76.06 ± 8.96^{bA}	43.83 ± 4.56^{dB}	64.47 ± 6.88^{dC}	43.00 ± 4.59^{cB}		28.66 ± 3.46^{aA}	15.42 ± 1.32^{eB}	36.11 ± 3.14^{dC}	21.19 ± 1.25^{cD}
**400**		63.44 ± 6.43^{aA}	48.40 ± 4.03^{aB}	51.31 ± 5.58^{aB}	54.69 ± 5.86^{dB}		29.05 ± 1.48^{aA}	22.19 ± 1.31^{aB}	22.15 ± 2.75^{aB}	23.74 ± 2.47^{cB}
**450**		65.60 ± 6.63^{aA}	48.63 ± 4.63^{aB}	46.28 ± 429^{aB}	49.41 ± 4.34^{aB}		28.45 ± 3.07^{aA}	21.83 ± 1.75^{aB}	16.36 ± 1.91^{eC}	25.06 ± 2.90^{cD}
**500**		39.50 ± 4.25^{cA}	63.06 ± 6.64^{cB}	92.54 ± 8.49^{cC}	38.79 ± 3.77^{dA}		18.46 ± 1.57^{dA}	25.01 ± 2.80^{bB}	29.37 ± 2.32^{bC}	17.30 ± 1.71^{bA}

Legend: EV: amniotic derived extracellular vesicle; CTR: control; VCL: curvilinear-line velocity; VSL: straight-line velocity. Different capital letters (A–D) in superscript indicate statistically significant differences ($P < 0.05$) between the time 0 and 4 hours incubation. Different lowercase letter (a–e) in superscript indicate statistically significant differences ($P < 0.05$) between different EV concentrations.

Supplementary Table S2. Sperm motility parameters evaluated by CASA System of sperm treated at different time with different concentration of AMSC-EVs, compared to CTR.

EV concentration ($x10^6$)/ml	VAP (μm/s)					AHL (μm)				
	0h	1h	2h	3h	4h	0h	1h	2h	3h	4h
**CTR**	53.85 ± 55.92	41.91 ± 4.08^{aA}	33.26 ± 3.27^{aA}	31.20 ± 3.69^{aA}	30.95 ± 3.12^{aA}	4.35 ± 3.73	3.87 ± 0.72^{aA}	3.70 ± 0.35^{aA}	3.79 ± 0.43^{aA}	3.62 ± 3.51^{aA}
**50**		17.79 ± 1.90^{bB}	36.07 ± 3.49^{aAB}	33.26 ± 4.66^{aA}	29.54 ± 3.26^{aA}		3.74 ± 0.95^{aA}	3.93 ± 0.68^{aA}	2.84 ± 0.97^{aA}	4.02 ± 2.83^{aA}
**100**		33.05 ± 3.86^{cC}	40.23 ± 4.40^{aB}	32.94 ± 3.46^{aA}	29.45 ± 3.43^{aA}		4.40 ± 0.48^{aA}	4.56 ± 0.77^{bA}	3.89 ± 0.94^{aA}	3.96 ± 3.35^{aA}
**150**		28.36 ± 1.96^{dC}	40.18 ± 4.70^{aB}	37.92 ± 3.45^{bA}	30.96 ± 3.52^{aA}		4.59 ± 0.94^{aA}	4.21 ± 0.72^{bA}	3.53 ± 0.98^{aA}	3.03 ± 4.08^{a}
**200**		28.50 ± 2.14^{dC}	40.54 ± 4.38^{aB}	33.76 ± 3.72^{aA}	29.21 ± 3.62^{aA}		4.70 ± 0.27^{aA}	4.72 ± 0.02^{bA}	3.32 ± 0.83^{aA}	3.76 ± 2.74^{aA}
**250**		29.17 ± 2.05^{dC}	48.61 ± 4.30^{bC}	35.44 ± 3.53^{aA}	29.08 ± 3.40^{aA}		3.16 ± 0.36^{aA}	4.06 ± 0.44^{bA}	3.41 ± 0.54^{aA}	3.08 ± 3.84^{aA}
**300**		43.81 ± 4.48^{eA}	40.65 ± 4.65^{aB}	35.10 ± 4.24^{aA}	30.55 ± 3.07^{aA}		5.69 ± 0.83^{bB}	3.34 ± 0.68^{aA}	3.60 ± 0.24^{aA}	3.07 ± 3.62^{aA}
**350**		48.75 ± 4.01^{eD}	40.95 ± 3.53^{aB}	35.06 ± 7.71^{aA}	30.50 ± 3.72^{aA}		3.75 ± 0.98^{aA}	3.40 ± 0.36^{aA}	2.95 ± 0.14^{aB}	3.59 ± 2.61^{aA}
**400**		39.98 ± 3.88^{cA}	41.94 ± 3.79^{aB}	35.79 ± 3.69^{aA}	30.10 ± 3.85^{aA}		3.23 ± 0.14^{aA}	3.60 ± 0.60^{aA}	3.41 ± 0.56^{aA}	3.30 ± 3.35^{aA}
**450**		44.75 ± 4.83^{eA}	36.32 ± 3.17^{aB}	35.75 ± 3.51^{aA}	30.15 ± 3.26^{aA}		3.84 ± 0.43^{aA}	3.80 ± 0.80^{aA}	3.21 ± 0.26^{aA}	3.96 ± 2.62^{aA}
**500**		64.07 ± 6.78^{fE}	36.18 ± 3.88^{aB}	32.62 ± 2.94^{aA}	30.71 ± 3.26^{aA}		4.65 ± 0.02^{aB}	3.65 ± 0.60^{aA}	5.86 ± 1.02^{bB}	3.50 ± 2.70^{aA}

Legend: EV: amniotic derived extracellular vesicle; CTR: control; VAP: average path velocity; AHL: amplitude lateral head. Different capital letters (A,B) in superscript indicate statistically significant differences ($P < 0.05$) between the time 0 and 4 hours incubation. Different lowercase letter (a,b) in superscript indicate statistically significant differences ($P<0.05$) between different EV concentrations.

Supplementary Table S3. Sperm motility parameters evaluated by CASA System of sperm treated at different time with different concentration of EVs, compared to CTR.

EV concentration ($x10^6$)/ml	LIN (%)					STR				
	0h	1h	2h	3h	4h	0h	1h	2h	3h	4h
**CTR**	44.58 ± 19.64	48.34 ± 3.01^{aA}	48.95 ± 2.60^{aA}	47.01 ± 1.21^{aA}	36.59 ± 1.20^{aB}	0.75 ± 0.28	0.86 ± 0.10^{aA}	0.84 ± 0.13^{aB}	0.86 ± 0.13^{aA}	0.78 ± 0.11^{aC}
**50**		44.82 ± 3.87^{bA}	46.46 ± 2.08^{aAB}	48.33 ± 1.61^{aB}	38.60 ± 2.43^{aC}		0.80 ± 0.14^{bA}	0.87 ± 0.11^{bB}	0.92 ± 0.19^{bC}	0.77 ± 0.11^{aD}
**100**		49.26 ± 4.58^{aA}	46.54 ± 4.14^{aA}	49.29 ± 2.22^{aA}	46.81 ± 1.91^{bA}		0.84 ± 0.11^{cA}	0.82 ± 0.12^{aB}	0.83 ± 0.11^{cB}	0.90 ± 0.10^{bC}
**150**		48.03 ± 4.77^{aA}	46.70 ± 4.96^{aA}	48.80 ± 2.34^{aA}	48.12 ± 2.21^{bA}		0.75 ± 0.12^{dA}	0.75 ± 0.19^{cA}	0.66 ± 0.19^{dB}	0.80 ± 0.10^{cC}
**200**		34.20 ± 3.67^{cA}	47.37 ± 4.94^{aB}	51.10 ± 2.64^{bB}	46.24 ± 1.55^{bB}		0.81 ± 0.13^{bA}	0.82 ± 0.10^{aA}	0.81 ± 0.16^{bA}	0.84 ± 0.13^{dB}
**250**		36.90 ± 2.90^{cA}	46.12 ± 4.93^{aB}	50.38 ± 2.42^{bB}	46.00 ± 1.68^{bB}		0.78 ± 0.16^{dA}	0.78 ± 0.15^{cA}	0.86 ± 0.11^{eB}	0.77 ± 0.14^{aC}
**300**		38.47 ± 1.41^{cA}	46.84 ± 3.34^{aB}	50.85 ± 1.47^{bC}	54.29 ± 2.30^{cD}		0.79 ± 0.18^{dA}	0.83 ± 0.11^{aB}	0.86 ± 0.10^{eB}	0.67 ± 0.11^{eC}
**350**		56.83 ± 2.17^{dA}	51.36 ± 4.36^{bB}	64.25 ± 2.16^{cC}	52.45 ± 2.41^{cB}		0.81 ± 0.16^{bA}	0.82 ± 0.16^{aA}	0.88 ± 0.19^{eB}	0.82 ± 0.18^{dA}
**400**		56.75 ± 2.75^{dA}	59.91 ± 2.35^{cAB}	54.49 ± 1.14^{dB}	54.37 ± 1.16^{cB}		0.87 ± 0.11^{aA}	0.87 ± 0.11^{dB}	0.85 ± 0.12^{aC}	0.88 ± 0.18^{bB}
**450**		50.32 ± 2.00^{eA}	52.68 ± 2.52^{bA}	41.07 ± 2.87^{eB}	47.39 ± 2.50^{bA}		0.83 ± 0.12^{bA}	0.79 ± 0.19^{cB}	0.82 ± 0.15^{bA}	0.81 ± 0.15^{cA}
**500**		44.28 ± 2.58^{fA}	49.13 ± 1.72^{aB}	37.09 ± 1.33^{fC}	48.82 ± 1.56^{bB}		0.82 ± 0.11^{bA}	0.82 ± 0.11^{aA}	0.72 ± 0.17^{fB}	0.89 ± 0.18^{bC}

Legend: EV: amniotic derived extracellular vesicle; CTR: control; LIN: linearity; STR: straightness. Different capital letters (A,B) in superscript indicate statistically significant differences ($P < 0.05$) between the time 0 and 4 hours incubation. Different lowercase letter (a,b) in superscript indicate statistically significant differences ($P<0.05$) between different EV concentrations
